# Migraine and heart: A reality check

**DOI:** 10.21542/gcsp.2023.20

**Published:** 2023-08-01

**Authors:** Jayesh Valecha, Harshwardhan Khandait, Anagha SK, Vasu Gupta, Sunita Kumawat, FNU Anamika, Rohit Jain, Dharti Dua

**Affiliations:** 1 Indira Gandhi Medical College, Shimla, Himachal Pradesh, India; 2 Trinitas Regional Medical Center/RWJ Barnabas Health, Elizabeth, NJ, USA; 3 Government Medical College, Thiruvananthapuram, India; 4 Dayanand Medical College and Hospital, Ludhiana, India; 5 Index Medical College Hospital & Research Center, Indore, India; 6 University College of Medical Sciences, New Delhi, India; 7 Penn State Milton S Hershey Medical Center, Hershey, PA, USA; 8 The Ohio State University College of Medicine, Columbus, Ohio, United States

## Abstract

Migraine is a common neurological disorder affecting 12% of the global population. The common risk factors are adolescent age, genetics, and female sex, and are triggered by hormonal fluctuations, emotional stress, sensory overload, weather changes, alcohol consumption, fasting, cheese, chocolate, smoked fish, yeast extract, cured meats, artificial sweeteners, food preservatives containing nitrates and nitrites, and sleep disturbances. Migraine with aura is associated with an increased risk of cardiovascular disease events, such as myocardial infarction, angina pectoris, and cardiac arrhythmias, and has recently been added to the QRISK3 cardiovascular disease prediction score. Population-based cohort studies have shown a significant association of migraine with aura and cardiac arrhythmias, most importantly atrial fibrillation. Patients suffering from migraine with aura are at an increased risk for cardiac arrhythmias; thus, it is essential to screen these patients for undiagnosed cardiovascular disorders.

## Introduction

Migraine is a complex cyclical brain disorder that is thought to be caused by dysfunction of sensory processing and homeostatic mechanisms. North America has the highest rate of migraine occurrence, followed by South and Central America, Europe, Asia, and Africa^1^. It is the second most frequent cause of disability among women worldwide after low back pain, thereby imposing a significant burden on individuals and society as a whole, as patients who experience acute attacks are less productive, resulting in absenteeism from work that can cause a consequential financial cost burden^2^. Assessing this cost burden using the Migraine Disability Assessment Scale (MIDAS) questionnaire revealed that worsening disability due to migraine causes a significant increase in healthcare utilization cost among American citizens^3^. The overall age-standardized prevalence is 12%, with a prevalence of 18% in women and 9% in men^4^. It is more prevalent among women than in men, and sex hormones are believed to play a key role in this discrepancy, as the variations in estrogen and progesterone have an effect on its etiology^5^. It is also associated with a strong genetic component; thus, the risk of developing migraine increases with a positive family history^6^. However, due to its complex genetic basis, no known inheritance pattern has been identified yet^7^. Several genome-wide studies have been conducted on previously identified genes that are expected to be involved in its pathophysiology, but no link between these genes and the onset of migraine has been discovered^8^.

Migraine can present with varying intensities of headache and is frequently accompanied by nausea, vomiting, photophobia, and phonophobia. The probable triggers for the development of migraine headaches include stress, hormonal changes during menstruation, ovulation, pregnancy, skipped meals, weather changes, excessive or insufficient sleep, odors (perfumes, colognes, petroleum distillates), exposure to light, alcohol ingestion, smoking, and certain foods^9^.

Migraines are classified as migraine with aura (MA), seen in at least one-third of patients, and without aura. Aura is defined as the sensory symptoms that may precede a migraine attack or present simultaneously. These can be visual (flickers of light or blind spots), auditory (tinnitus), or sensory (numbness or tingling)^10^. The widely accepted theory for the pathophysiology of MA is cortical spread depression, according to which there is a spread of neuronal and glial depolarization across the cerebral cortex that in turn activates trigeminal afferents, which alter the pain-sensitive meninges in an inflammatory manner through central and peripheral reflex pathways to produce migraine headaches^11^. Patients with MA have been associated with an increased risk of cardiovascular diseases such as ischemic heart disease^12^ and cardiac arrhythmias, most commonly atrial fibrillation, which may lead to ischemic stroke or transient ischemic attack (TIA)^13^.

Many studies have shown that migraine, especially migraine with aura, is associated with patent foramen ovale (PFO), which is supported by the finding that closure of PFO significantly reduces the frequency and severity of migraines in some individuals^14^. A meta-analysis of case-control studies found that PFO is associated with a 3.4-fold prevalence of migraine with aura and a 2.5-fold prevalence of migraine with or without aura^15^. The QRISK risk calculator, which is used to predict 10-year cardiovascular disease, was recently updated in 2018 as QRISK3. This updated algorithm now includes the diagnosis of migraine including all the subtypes as a potential risk factor for cardiovascular mortality for individuals 25 to 84 years of age^16^. While developing and validating the QRISK3 predictor, it was found that migraine increased the risk of cardiovascular events by 36 percent in females and 29 percent in males^17^.

**Figure 1.**
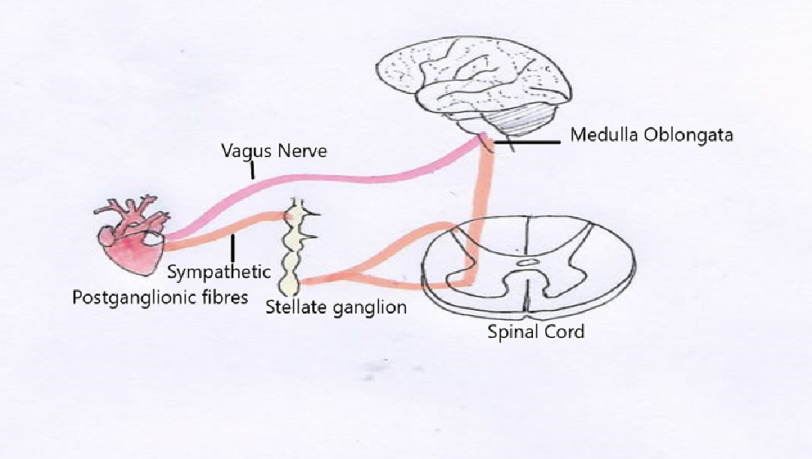
Autonomic innervation to the heart.

Considering that migraine is associated with increased cardiovascular risks, in this article, we will review the pathophysiology of migraine, its relation to cardiac arrhythmias and PFO, as well as the diagnostic and treatment options available for both migraine headaches and cardiac arrhythmias.

## Pathophysiology

Autonomic innervation plays an important role in maintaining heart rate, rhythm, and contractility. The cardiac nervous system can be divided into intrinsic and extrinsic components. The pacemaker cells and atrial stretch receptors of the heart comprise the intrinsic component of the cardiac ANS, while the sympathetic and parasympathetic components make up the extrinsic cardiac nervous system^18^. Sympathetic innervation begins with excitatory impulses sent from the rostral ventrolateral medulla neurons to the anteromedial cell column of the T1-L2 spinal segments, where cardiac preganglionic neurons are located. These preganglionic axon terminals project primarily to the stellate and paravertebral ganglia, which comprise the main postganglionic sympathetic supply^19^. Parasympathetic innervation of the heart originates in the nucleus ambiguus and the dorsal motor nucleus of the vagus nerve in the brainstem (Figure 1). These preganglionic neurons send axons that are carried by the vagus nerve and synapse in the cardiac ganglia, where they release ACh, resulting in a decreased heart rate by decreasing SA node and AV node conduction^20^.

One of the main characteristics of migraine is dysfunction of the autonomic nervous system (ANS)^21^. As a result, migraine sufferers experience a wide range of symptoms, including sinusitis-like symptoms such as nasal congestion, rhinorrhea, nausea, vomiting, and cutaneous vasoconstriction^22^. In patients with migraine, this reversible imbalance in the autonomic innervation of the heart can lead to disturbances in cardiac physiology. Cardiac arrhythmias can result from disturbances in the sympathetic and/or parasympathetic systems of the ANS. Atrial fibrillation (AF) is assumed to result from simultaneous stimulation of the sympathetic and parasympathetic nervous systems, whereas ventricular fibrillation or ventricular tachycardia is thought to result from enhanced sympathetic stimulation^23^. Patients with migraine without risk factors for coronary artery disease can have electrocardiographic alterations during a migraine attack due to coronary vasospasm secondary to either increased activity of the sympathetic nervous system or serotonin [5HT]1B/1D agonist drugs treatment of migraine. This leads to ischemia distal to the spasmodic segment, which can result in acquired prolongation of the QT interval, setting the stage for malignant arrhythmias^24,25^. 

The underlying pathophysiology of the association between Migraine and PFO is still based on hypotheses and possible mechanisms, including microembolus-triggered cortical spreading depression, the vasoactive substance hypothesis, genetic factors, and impaired cerebral autoregulation. PFO may trigger cortical spreading depression by allowing the passage of microemboli in the venous circulation into the arterial system. Right-to-left shunting (RLS) through PFO leads to decreased blood oxygen saturation and hypoxia, which increases the expression of plasminogen activator-1 and results in the inhibition of fibrinolysis, thus increasing the possibility of microembolization. This can result in hypoperfusion and brain injury, triggering cortical spreading depression that may cause migraine attacks. The vasoactive substance hypothesis states that *via* PFO, certain vasoactive substances such as serotonin, nitric oxide, endothelin, and kinin can bypass the pulmonary circulation without being metabolized and pass directly through the blood–brain barrier to cause migraine. Another hypothesis states that dysfunctional autoregulation of intracranial microvessels may be involved and that genetic factors may also play an important role^26,27^.

In one of the cohort studies conducted in the Danish population, it was found that migraine with visual aura was associated with an increased risk of AF compared to no headache (hazard ratio 1.30, 95% confidence interval 1.03–1.62) as well as when compared to migraine without visual aura (hazard ratio 1.39, 95% confidence interval 1.05–1.83)^28^. Similar results were found in another cohort study, Atherosclerosis Risk in Communities. After adjustment for multiple confounders, migraine with visual aura was associated with increased risk of AF compared to no headache (hazard ratio 1.30, 95% confidence interval 1.03–1.62) and migraine without visual aura (hazard ratio 1.39, 95% confidence interval 1.05–1.83)^13^.

Certain electrocardiographic (ECG) changes have been found to be predictors of arrhythmia in patients with migraine. Corrected QT interval (QTc) dispersion has been suggested as an electrocardiographic index that reflects the physiological variability of regional ventricular repolarization. In a study by Duru et al., higher QT interval parameters (QTc interval, maximum and minimum QT duration, and QTc dispersion) and P-wave dispersion on ECG monitoring were found during migraine attacks than during pain-free periods. QT dispersion was defined as the difference between the longest QT interval (QTmax) and shortest QT interval (QTmin) and P-wave dispersion, defined as the difference between the maximum P-wave duration and the minimum P-wave duration^29^. Changes in T-waves have also been studied as predictors of ventricular arrhythmias, and in a cross-sectional, observational study, T-wave peak-to-end (Tp-e) interval and Tp-e/QT ratio were significantly increased during migraine attacks compared with those during attack-free periods and can be used to predict ventricular tachyarrhythmias and cardiovascular mortality^25^. As these patients have an increased risk of cardiovascular morbidity and mortality, timely diagnosis and treatment are important.

## Diagnosis and treatment

Migraine with aura is a clinical diagnosis. As per the International Classification of Headache 3 (ICHD 3) guidelines, diagnostic criteria involve at least two attacks with: one or more fully reversible aura symptoms of visual, sensory, speech, motor, brainstem, and retinal in addition to at least three from the following list: at least one aura symptom spreading gradually over >five minutes; two or more symptoms occurring in succession; each aura symptom lasting 5–60 min; at least one aura symptom being unilateral; at least one aura symptom being positive; aura accompanied, or followed within 60 min, by headache^30^. 

The acute treatment depends on the severity of the attacks. For mild to moderate attacks, defined as episodes of migraine with no associated functional limitation, nonsteroidal anti-inflammatory drugs (NSAIDS) such as Ibuprofen, Naproxen, and acetaminophen^31^ are first-line agents as they are effective and inexpensive. For moderate to severe attacks (i.e., limiting work, social, or family activities) and in patients in whom NSAIDS have failed in the past, triptans (sumatriptan and rizatriptan) or a combination of triptans and naproxen are preferred^32^. As triptans are vasoconstrictors, they may increase the risk of serious ischemic events in patients with underlying cardiovascular disease (CV) or risk factors for CV disease. Therefore, triptans are contraindicated in patients with certain CV diseases, such as coronary artery disease, coronary artery vasospasm, and peripheral artery disease^33^. If contraindications to triptans exist, newer agents, such as calcitonin gene-related peptide (lasmiditan), are an alternative. In attacks lasting >72 h, NSAIDS, such as ketorolac with intravenous fluids, can be used.

The associated aura symptoms of nausea and vomiting are managed with oral antiemetics such as ondansetron. In severe cases of nausea/vomiting, when oral agents cannot be used, subcutaneous sumatriptan and nasal sumatriptan are alternatives. While acute migraine treatment mainly targets symptom relief after migraine has begun, prophylactic therapy focuses on preventing migraine attacks. Patients with recurrent migraine attacks usually begin prophylactic therapy. Chronic preventive therapy includes beta-blockers (metoprolol, propranolol, and timolol), antidepressants (venlafaxine and amitriptyline), and anticonvulsants (valproate and topiramate) and is indicated in patients with frequent or long-standing migraine, diminished quality of life, contraindications/failure of acute therapy, and adverse effects of acute therapy^34^.

Small molecule calcitonin gene-related peptide (CGRP) antagonists, also known as “gepants” such as rimegepant^35^ and ubrogepant^36^, have been used for acute treatment of migraines in patients with cardiovascular history as these do not cause vasospasm. CGRP monoclonal antibodies, such as fremanezumab^37^, galcanezumab^38^, and erenumab^39^, have been recently approved by the FDA for migraine prevention.

Migraine with aura has been associated with cardiac arrhythmias, including atrial fibrillation, sinus arrhythmia, atrial premature contraction, ventricular premature contraction, increased QTc dispersion, p wave dispersion, sinus bradycardia, and prolonged PR interval^22^. Cardiac arrhythmias are diagnosed using a 12 lead ECG and treatment depends on the type of arrhythmia. AF is a major cause of stroke in patients with migraine with aura. A study by Gollion et al. reported that atrial fibrillation accounted for 10.34% of stroke cases in migraine with aura patients^40^. Thus, early diagnosis and treatment are essential.

The core components of AF therapy include rate control and rhythm control if AF remains symptomatic despite adequate rate control and anticoagulation therapy. Rate control was achieved with beta blockers and calcium channel blockers. Rhythm control is typically achieved using amiodarone^41^. Anticoagulation is achieved with warfarin or direct oral anticoagulants (DOACs), such as apixaban and rivaroxaban, based on the CHADSVASc score. Anticoagulation is important for preventing stroke and other embolic complications^42^. DOACs and fondaparinox are preferred in patients with heparin-induced thrombocytopenia. Migraine is known to increase the risk of intracranial hemorrhage, and the use of anticoagulants for AF can potentiate this risk^43^.

Asymptomatic individuals with atrial premature contractions do not need any therapy, whereas symptomatic patients are counselled to avoid precipitants, such as alcohol and caffeine. Persistent symptoms are treated with beta blockers or catheter ablation^44^. For patients with premature ventricular contractions, risk factors and precipitants, such as alcohol, caffeine, uncontrolled hypertension, and thyroid abnormalities, are corrected. In high-risk patients, such as those with pre-existing cardiac structural or conduction system disease, management with beta blockers, antiarrhythmics, or catheter ablation is preferred^45,46^. Ventricular tachycardia (VT) management depends on the patient’s hemodynamic status and whether the VT is sustained or non-sustained^47^. For hemodynamically stable patients, rate control with beta-blockers and calcium channel blockers is the initial strategy, whereas rhythm control, particularly with amiodarone, is performed if the symptoms are uncontrolled. In unstable patients, electrical cardioversion/defibrillation is the initial step. 

Numerous studies have shown that migraine with aura is associated with an increased risk of ischemic stroke^48^. One suggested hypothesis is migraine-induced autonomic dysfunction, which can increase the risk of cardiac arrhythmias and subsequent ischemic stroke. Prior studies have shown migraine with aura to be an independent risk factor for new-onset AF^13^. Thus, screening patients for paroxysmal AF in patients with migraine with aura might be reasonable to prevent stroke complications. Since there is no increased risk of sudden cardiac death in migraine patients, guidelines for routine arrhythmia screening are not defined, and more research is needed^21^. 

## Conclusion

Migraine is a known risk factor for ischemic stroke, and its recent association with cardiac arrhythmias and atrial fibrillation may provide a clearer picture of this association. People suffering from migraine with aura are at a higher risk of developing atrial fibrillation as compared than those without aura. Although a significant association has been demonstrated, the underlying mechanisms remain unclear. Recently, the QRISK3 predictor has included migraine as a risk factor for overall cardiovascular diseases; however, the United States of America has yet to adopt this new risk factor. It is vital to remain vigilant and actively screen for atrial fibrillation in patients suffering from chronic migraines, as it can substantially decrease the burden of thromboembolic stroke and reduce the incidence of cerebrovascular events and mortality. Formal guidelines and protocols are needed to achieve this, and more research is required to determine the exact mechanism by which migraine leads to atrial fibrillation. 

## Abbreviations

Migraines with Aura (MA), Migraine Disability Assessment Scale (MIDAS), Transient Ischemic Attack (TIA), Autonomic Nervous System (ANS), Atrial Fibrillation(AF), Electrocardiogram (ECG), Corrected QT interval (QTc), T-wave peak-to end (Tp-e), International Classification of Headache 3 (ICHD 3), Nonsteroidal Anti-Inflammatory Drugs (NSAIDS), Calcitonin Gene-Related Peptide (CGRP), Direct oral anticoagulants (DOACs), Ventricular tachycardias (VT).

## Author statement

**Conceptualization**: Jayesh Valecha, Harshwardhan Khandait, Anagha SK, Vasu Gupta

**Supervision**: Rohit Jain, Dharti Dua

**Writing - Original Draft Preparation**: Jayesh Valecha, Harshwardhan Khandait, Anagha SK, Vasu Gupta, Sunita Kumawat, FNU Anamika

**Writing - Review & Editing:** Sunita Kumawat, FNU Anamika, Rohit Jain, Dharti Dua
